# Medical and Physical Intelligence

**Published:** 1804-12-01

**Authors:** 


### MEDICAL AND PHYSICAL INTELLIGENCE.

### [ FOREIGN AND DOMESTIC. ]

---

The "Report of a Medical Committee on the Cases of supposed Small-pox after Vaccination, which occurred in Fullwood's Rents, Holborn, in August and September, 1804, with an Account of some subsequent Inoculations," having been published as we were committing our last sheet to the press, we are anxious to give our readers the earliest, though it must be but a short and most hasty notice of it. The following is the commencement of the Report.

"The cases of two children in Fullwood's Rents, Holborn, said to be cases of small-pox after vaccination, having very much engaged the attention of the public, as well as that of the faculty, it was thought expedient by a numerous body of medical practitioners, met at the house of Messrs. Morgan and Wigham, in Holborn, on Wednesday, October the 3d. 1804, to form a Committee for the purpose of investigating the minute particulars of each case, and of making some experiments, calculated to remove every doubt on the subject.

"A committee was accordingly formed, consisting of the following gentlemen:— Dr. Gower, Dr. Ash, Dr. Pemberton, Dr. Willan, Dr. Temple, Dr. Clarke, Dr. Croft, Dr. Yelloly, Dr. Skey, Mr. Hurlock, Mr. Addington, Mr. Pears, Mr. Morgan, Mr. Wigham."

As the objects of attention had both been vacciolated at the Small-Pox Hospital, a correct copy of the particulars respecting their inoculation was obtained from the register of that Institution, and is given in the Report. The cases of these two children, under the eruptions in question, are first described. With matter taken from the younger child, a series of inoculations was instituted. Cases 3 and 4 give the result of the first experiments. Cases 5, 6, and 7 were inoculated from 3; and 8 was afterwards inoculated from 7; all of which were considered to have the variolous disease produced by the inoculations. Case 8 was even re-inoculated with variolous matter, "with a wish to satisfy some practitioners who could not persuade themselves that the eruption on the child from whom the matter was taken was the small-pox, but who supposed it might be either generically different from the variolous eruption, or some modification of it that would not emancipate the constitution from being again affected with the genuine disease." Case 9 was inoculated from 6; but having been vacciolated at the Small-pox Hospital three years ago, he only was affected similarly to the cases of Mr. Crieghton and others, who who have subjected their vacciolated patients to the test of variolous inoculation. As he slept in the same bed with 5 and 6, his brothers, while they were under inoculation, the Committee consider him to have resisted both the contagion and inoculation of small-pox.

The following is the conclusion of their Report:

" After having faithfully reported the particulars of the investigation proposed, (page 3), the Committee begs leave to observe, that there seems no reason to question the regular progress of the vaccination in Nancy and Mary Hodges, nor the existence of the small-pox more than two years afterwards in the latter, there being no material variation from the usual course of symptoms, either in the disease of Mary Hodges, or in the cases of inoculation with matter taken from her pustules.—The Committee, however, feels it a duty to remark, that the above facts are not to be considered as militating against the general practice of vaccination. Some well authenticated, though rare, cases have been stated, in which the natural small-pox occurred twice in the same person. A few other instances are recorded of persons, who, after having undergone the inoculated small-pox, nevertheless took the disease by infection: yet these cases were not deemed conclusive against the advantages of variolous inoculation, nor do they seem to have impeded its progress.

" In every country where European science is diffused, the general preventive power of vaccine inoculation with regard to the small-pox, has been fully ascertained, and cannot now be affected by the result of a few detached cases, which, by future observations and experiments, may be accounted for satisfactorily—The Committee, therefore, with one accord, subscribes to the established opinion, that if vaccination were universally adopted, it would afford the means of finally exterminating the Small-pox."

---

Mr. Buchholz has made some experiments on the hydrargyrus muriatus mitis (mercurius dulcis). The common method of preparing this salt is by sublimation of seven parts of mercury and three parts of oxygenated muriat of mercury; the precipitation adopted by Scheele having been laid aside, because apothecaries are of opinion that this production on being mixed with lime water or alkaline solutions does not sufficiently blacken those substances. The author, therefore, examines whether these productions be really different from one another. For this purpose equal parts of mercury and of nitric acid are mixed together, and left for some time in the cold; it is then exposed to a gentle heat in a sand bath till it begins to boil, after which the liquor is poured, whilst hot, into a solution of muriat of soda, that contains equal parts of this alkali, and mercury. The precipitate obtained by water saturated with sal ammoniac or muriat of ammonia, is boiled and carefully washed; a copious production is obtained; and the author proves, by a series of experiments, that it does not differ from that obtained by corrosive sublimate; this method also appears to him more advantageous, and less dangerous than that by sublimation.

Mr. SCHMIDT has made the following experiment on the decomposition of sulphat of pot-ash and of sulphat of soda, in the dry way, by means of quick lime. On igniting for half an hour a mixture of three parts of quick lime and two parts of sulphat of pot-ash, afterwards dissolving the mixture and evaporating the liquor, carbonat and sulphat of lime will be precipitated, whilst a small quantity of carbonat of pot-ash remains in the water. He made the same experiments with sulphat of soda, the results of which were more successful, and the decomposition more complete. It is therefore certain that the decomposition of these two sulphats takes place by quick lime, but that these processes do not appear to be very profitable.

---

*Mr. Dupuytren on the Formation of the Larynx in Eunuchs.*

There exists in the animal economy several instances of the influence which takes place between organs that are not contiguous to one another; one of the most remarkable instances of this kind is the sympathy which subsists between the testicles and the organs of voice. The larynx is observed to develope itself in several animals during the rutting season, and the smallness of the larynx, the narrowness of the glottis, and the shrill voice, coincide with the state of the inactivity which the testicles show before the approach of puberty. This period however being arrived, the organs for seminal secretions are developed, and become active, while, at the same time, the larynx rapidly increases in males, and the voice takes that grave sound which makes one of the characters of virility. But, on the contrary, when the testicles are cut away before this period, the source of the phenomena which characterise it vanishes, and the organs of voice remain in a state of imperfection. Mr. Dupuytren has lately had an opportunity of confirming the justness of the observation by dissecting the larynx of a man who had been castrated in his infancy, as he found this organ to be one third less than it is met with in most men of the same age and habit. The glottis was very narrow, and all the organs of voice rather resembled those of a woman, or a youth before the period of puberty.

---

*On Heat disengaged by the Compression of Air.*—A very curious experiment has been lately repeated before the National Institute. On forcibly and rapidly compressing the air in the pump of a wind gun, a considerable heat is disengaged at the first push of the piston, sufficient to inflame a piece of tinder which is placed within the pump. When the body of the pump is terminated with a mobile end, closely screwed to it, having in its centre a strong glass lens, a vivid and brilliant light will be seen, which is suddenly disengaged by the first push of the piston. This discovery is said to have been accidentally made by a workman in the manufactory of fire-arms, at St. Etienne. It has been successfully repeated by Mr. Mollet, of Lyons, and certainly deserves the notice of naturalists, as it may tend to explain the nature of light and heat.

Mr. J. C. SAUNDERS, Demonstrator of Practical Anatomy at St. Thomas's Hospital, has lately published a Proposal for the Establishment of a Dispensary for the Relief of Persons afflicted with Diseases of the Eye and the Ear; which has been recommended to the public by the signatures of all the physicians and surgeons of St. Thomas's and Guy's Hospitals. The following is the plan upon which it is to be conducted.

1. That a Dispensary be opened for the benefit of the poor, who are afflicted with diseases of the eye and the ear, where they may apply, and obtain medicines gratis.

2. That the Dispensary be central in its Situation, and contain two beds for the reception of patients who undergo the operation for the cataract, or any other operation of the eye requiring minute care.

3. That the Charity may be established at as little expence as possible, it is suggested that a house of a very moderate rent, will be sufficient for all the purposes of this charity.

4 That a person contributing an annual subscription of One Guinea, be a Governor, and have the right of recommending, and keeping under the care of the charity, one out-patient; or two, if his subscription amounts to Two Guineas.

5. That Persons afflicted with cases, which make it necessary that they should be received into the house, shall be received according to priority of recommendation.

---

A second edition of Dr. TROTTER's Essay, Medical, Philosophical, and Chemical, corrected and enlarged, is now in the press, and will be published in the course of the ensuing month.—French and German translations of this Essay are announced at Paris and Vienna.

